# Dynamics of clinical *Klebsiella pneumoniae* strains over the COVID-19 pandemic in Qingdao, China

**DOI:** 10.1128/aem.00706-26

**Published:** 2026-06-29

**Authors:** Shengyao Wang, Peng Lin, Peina Du, Wenya Su, Sensen Lv, Lei Dong, Peikun Teng, Xiudi Han, Xu Zheng, Yujie Wang, Ling Li, Xueyun Geng, Mengge Zhang, Kailin Wang, Shengying Li, Mingyu Wang, Xuedong Liu

**Affiliations:** 1 Qingdao Municipal Hospital, School of Pharmacy, Qingdao University 12593, Qingdao, Shandong, China; 2 Department of Respiratory and Critical Care Medicine, Qingdao Municipal Hospital 12648, Qingdao, Shandong, China; 3 State Key Laboratory of Microbial Technology, Microbial Technology Institute, Shandong University 520252, Qingdao, Shandong, China; 4 Clinical Laboratory, BGI Genomics, Qingdao, Shandong, China; 5 Department of Clinical Laboratory, Qingdao Municipal Hospital 12648, Qingdao, Shandong, China; Centers for Disease Control and Prevention, Atlanta, Georgia, USA

**Keywords:** *Klebsiella pneumoniae*, whole-genome sequencing, COVID-19, co-occurrence network analysis, antimicrobial resistance, virulence factors

## Abstract

The dynamics of multidrug-resistant pathogens in response to large-scale societal and environmental disruptions remain poorly understood. Here, we investigated 528 *Klebsiella pneumoniae* clinical isolates collected from a tertiary hospital in Qingdao, China, spanning 2016 to 2023, with a primary focus on 2018–2023, across pre-, during-, and post-COVID-19 pandemic periods. All isolates underwent whole-genome sequencing. We identified SL258 and SL23 as dominant sequence lineages, with prevalent serotypes including K1 and O1. A wide range of resistance and virulence determinants were detected, particularly *ybt* genes, which showed strong lineage-specific associations. The COVID-19 pandemic coincided with a transient expansion of SL258 and an overall increase in antimicrobial resistance levels, followed by post-pandemic diversification of resistant clones. The structural complexity of the *K. pneumoniae* population decreased during the pandemic but rose significantly afterward, with the emergence and expansion of multiple previously rare multidrug-resistant (MDR) lineages. A serotype shift within SL258, from K25/O5 to K64/O2a, was also observed. These findings highlight the selective pressures exerted by hospital-based infection control strategies and antimicrobial usage patterns during the pandemic, which may have shaped the persistence and replacement of dominant MDR clones. Our study underscores the importance of genomic surveillance in tracking resistance evolution and pathogen adaptation in clinical environments under crisis conditions.

**IMPORTANCE:**

Multidrug-resistant *Klebsiella pneumoniae* (MDR-KP) is a critical global threat, linked to longer hospital stays, higher treatment costs, and worse patient outcomes. The COVID-19 pandemic placed unprecedented pressure on healthcare systems, altering antibiotic use and infection control practices that may have reshaped MDR-KP populations. Our 8-year surveillance of clinical *K. pneumoniae* strains in Qingdao reveals how pandemic-related disruptions drove shifts in lineage dominance, antimicrobial resistance profiles, and serotype distribution. These findings highlight the vulnerability of clinical microbial ecosystems to large-scale public health crises and underscore the need for sustained genomic surveillance. By establishing a longitudinal institutional baseline for MDR-KP dynamics, our work provides actionable insights to guide local infection control and antimicrobial stewardship, helping to mitigate the spread of resistant pathogens in similar clinical settings during future health emergencies.

## INTRODUCTION

*Klebsiella pneumoniae* is a key clinical pathogen that causes a broad spectrum of diseases including pneumonia and bacteremia (1). It is among the most frequently identified infectious bacterial agents in clinical settings and ranks second in prevalence among all bacterial isolates in China’s CHINET surveillance program(2). *K. pneumoniae* infections can be associated with high mortality rates; bloodstream infections caused by this pathogen may result in mortality rates as high as 40% (3). Furthermore, *K. pneumoniae* has been classified in the critical priority group on the World Health Organization Bacterial Priority Pathogens List for 2024, due to its role as a member of carbapenem-resistant Enterobacterales.

One problem that is holding back *K. pneumoniae* infection treatment is antimicrobial resistance (AMR). The development of AMR is a major threat to humanity, with 4.71 million deaths associated with bacterial AMR in 2021 globally and 8.22 million deaths projected by 2050 (4). In the case of *K. pneumoniae*, development of carbapenem resistance has increased rapidly over the past two decades. The CHINET data indicate that the resistance rates of *Klebsiella pneumoniae* to imipenem and meropenem in China have increased from 3.0% and 2.9% in 2005 to 22.5% and 23.6% in 2023 (2).

*K. pneumoniae* strains can be classified based on their genomic sequences using gene panels included in traditional Multilocus Sequence Typing (MLST) (5) or more recent core genome MLST (cgMLST) schemes (6). Certain sequence types (STs), such as ST23 *K. pneumoniae* strains, have been generally known as hypervirulent strains, whereas some STs like ST11 are recognized as multidrug-resistant (MDR) strains. Hypervirulence and multidrug resistance are not mutually exclusive, as hypervirulent MDR strains are frequently reported (7).

The COVID-19 pandemic between 2019 and 2022 led to tremendous life losses and global economic slowdown (8). It is also the first global-scale attempt to use social distancing and self-protection (such as facial masks) to reduce disease transmission in human history, with massive success in controlling the disease (9). This has also cut off the transmission of other pathogens between individuals, creating isolated ecosystems for pathogens, rather than having the whole human population as a connected community. However, knowledge on how this isolation impacted the development and evolution of pathogen communities in the human population is still limited.

In this work, a surveillance was carried out on the whole-genome sequences, sequence types, serotypes, AMR, and virulence on 528 *K. pneumoniae* strains collected before, during, and after the COVID-19 pandemic in Qingdao, China. This work aims at filling the knowledge gap on how the COVID-19 pandemic impacted the *K. pneumoniae* community, which also provides insight on how we can do better in the likely inevitable next pandemic.

## MATERIALS AND METHODS

### Clinical isolates and antimicrobial susceptibility tests

All *K. pneumoniae* strains were retrieved from Qingdao Municipal Hospital bacterial connection and were subsequently purified on lysogeny broth (LB) plates. For temporal analysis, isolates were categorized into three periods: pre-pandemic (prior to 2019), during-pandemic (2020–2022), and post-pandemic (defined exclusively by isolates collected in 2023). AMR phenotypes were determined using the VITEK2 System (bioMérieux, Inc., Durham, NC, USA). Both broth dilution and disk diffusion methods were used for validation and confirmation, following the Clinical and Laboratory Standards Institute standard (10), except for polymyxin and tigecycline. Polymyxin susceptibility was assayed following the EUCAST standard (11). Tigecycline susceptibility was assayed following the FDA/NMPA standard (12). *Escherichia coli* ATCC 25922 was used as the quality control strain.

### Whole-genome sequencing

The DNA was extracted by bacterial DNA extraction kit, and second-generation sequencing was performed with an MGISEQ-2000 system (MGI Tech Co., Ltd., Shenzhen, Guangdong, China) at PE150 mode. Fastp v0.23.4 was used for read trimming and read-level quality control (13). Genome assembly was performed using SPAdes v3.13.1 (14). Quality control was done with QUAST v5.0.2 (15), BUSCO v5.2.2(16), and CheckM2 v1.0.1 (17).

### Genome analysis

Bacterial taxonomy identification was performed using GTDB-tk v2.1.1 (18). For genomic characterization, STs, serotypes, and virulence genes were identified with Kleborate v2.3.2 (19, 20) as the primary screening tool. To achieve higher resolution for phylogenetic analysis, cgMLST was performed using Pathogenwatch (https://pathogen.watch/), with isolates further categorized into sublineages (SLs) and clonal groups (CGs) based on core-genome allelic profiles. Antibiotic resistance genes (ARGs) were identified using AMRFinder v3.11.1 with database version 2023-04-17.1 (21).

### Phylogenetic analysis

The single-nucleotide polymorphisms were identified and combined using snippy v4.6.0 (https://github.com/tseemann/snippy.git). Gubbins v3.3.1 was then used for deduplication (22). Concatenated sequences of filtered polymorphic sites conserved in all the isolates were used to perform phylogenetic analysis using the maximum likelihood method by IQ-Tree v2.3.6 (23). Finally, the phylogenetic trees were visualized by iTOL v7.0 (24).

### Co-occurrence network analysis

Pearson correlation was calculated between each pair of features in the analysis.

Correlations were limited to strong connections (*R* > 0.6) to avoid false positive discoveries. Features with fewer than five members were also excluded. The co-occurrence network was constructed with Gephi v0.10.1.

### Statistical analysis of resistance trends

Longitudinal changes in antibiotic non-susceptibility rates during the study period were evaluated using the Cochran-Armitage trend test. For this analysis, clinical isolates were categorized as either susceptible or non-susceptible (including resistant, intermediate, and susceptible-dose dependent strains). The year of isolation was treated as an ordinal variable to assess the significance of resistance trajectories. Annual non-susceptibility rates and their corresponding 95% confidence intervals were calculated using the Wilson score method via the R package binom (version 1.1-1.1). All statistical analyses and trend visualizations were performed using R version 4.5.1 with the DescTools and ggplot2 packages. A two-tailed *P* < 0.05 was considered statistically significant.

## RESULTS

### Typing, virulence factors, and AMR characteristics of *K. pneumoniae* strains

**Fig 1:**
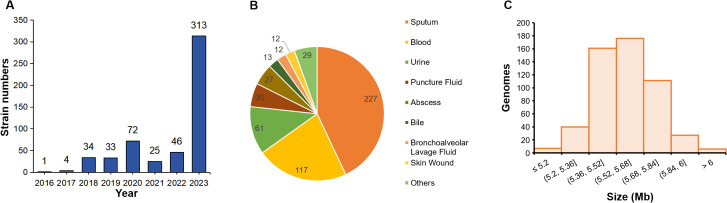
Collected *K. pneumoniae* samples. (**A**) Collected strain numbers per year; (**B**) sources of collected strains; (**C**) genome sizes determined with whole-genome sequencing.

Five hundred and twenty-eight *K. pneumoniae* strains were retrieved from the clinical bacterial strain collection of Qingdao Municipal Hospital (Table S1), in order to characterize the dynamics of this important pathogen during the COVID-19 pandemic. These strains primarily spanned a 6-year period from 10 January 2018 to 15 October 2023, with the addition of five sporadic isolates from 2016 (*n* = 1) and 2017 (*n* = 4) (Fig. 1A). This timeframe covers 2016–2019 before the pandemic (pre-pandemic, 72 strains), 2020–2022 during the pandemic (during-pandemic, 143 strains), and 2023 after the pandemic (post-pandemic, 313 strains). The majority of the strains were isolated from sputum, blood, and urine samples (Fig. 1B). High-throughput second-generation sequencing was performed with these strains, generating whole genomic sequences. Most genomes fall in the size range of 5.36 Mb and 5.84 Mb (Fig. 1C), in accordance with common genome sizes of *K. pneumoniae*.

**Fig 2:**
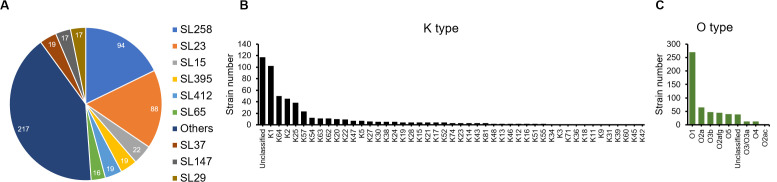
Typing of *K. pneumoniae* strains. (**A**) Distribution of major *K. pneumoniae* sublineages; (**B**) frequency distribution of K serotypes in *K. pneumoniae* strains; (**C**) frequency distribution of O-serotypes in *K. pneumoniae* strains.

*K. pneumoniae* strains were typed with MLST and cgMLST methods, leading to identification of clonal groups (CGs), sublineages (SLs), and sequence types (STs) (Table S1). The 528 strains belong to 126 STs, 110 CGs, and 91 SLs. The two classification methods showed good correlation. Therefore, the previously acknowledged correlation of sequence types with phenotypic features is still valid, although cgMLST uses a much larger panel of core genes for classification. The typing of strains on the SL level considers the relatedness of different CGs and STs and is therefore a more stable method to analyze sequence type changes. We subsequently used SLs as methods for sequencing typing. As shown in Fig. 2A, the two most frequent sequence types found are the MDR SL258 (containing both ST11 and ST258) and the hypervirulent SL23. Serotyping of collected strains based on their surface antigens showed that the strains belong to 46 K-types and 8 O-types, with serotype K1 and O1 being the two most frequent serotypes (Fig. 2B through C).

**Fig 3:**
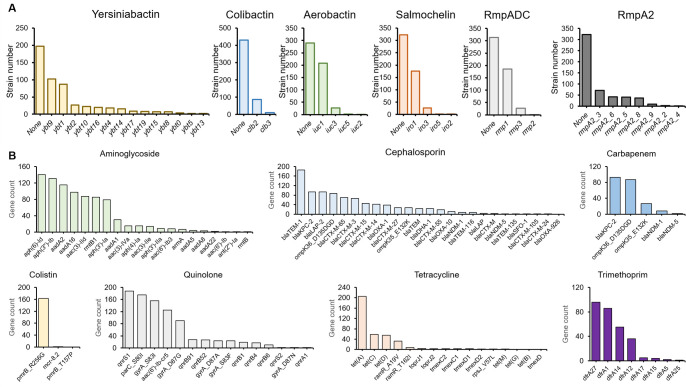
Virulence factors and AMR genes. (**A**) Frequency distribution of virulence factors in *K. pneumoniae* strains; (**B**) frequency distribution of AMR genes in *K. pneumoniae* strains.

The presence of virulence factors and ARGs was studied with whole-genome sequence annotation (Fig. 3). Virulence factors were widespread for the immune escape facilitating *ybt* genes (62.7%), the genotoxin *clb* genes (17.4%), the siderophore *iuc* (45.1%) and *iro* (38.8%) genes, and the hypermucoid *rmp* (40.5%) and *rmpA2* (39.0%) genes (Fig. 3A), which is higher than what was reported in a global surveillance containing 9,705 non-redundant *K. pneumoniae* strains (16), suggesting high virulence levels. For ARGs (Fig. 3B and Table S2), several observations can be made: (i) aminoglycoside resistance genes associated with mobile genetic elements were common, (ii) the most prevalent cephalosporin and carbapenem resistance genes are *bla*_TEM-1_ and *bla*_KPC-2_, (iii) mobile polymyxin-resistant *mcr-8.2* was found in two strains, and (iv) the transferrable *toprJ-tmexCD* leading to tigecycline resistance (25) was identified in seven isolates (1.3%). Specifically, the *tmexCD1-toprJ1* cluster was found in four isolates, while the *tmexCD2-toprJ2* cluster was detected in three isolates.

**Fig 4:**
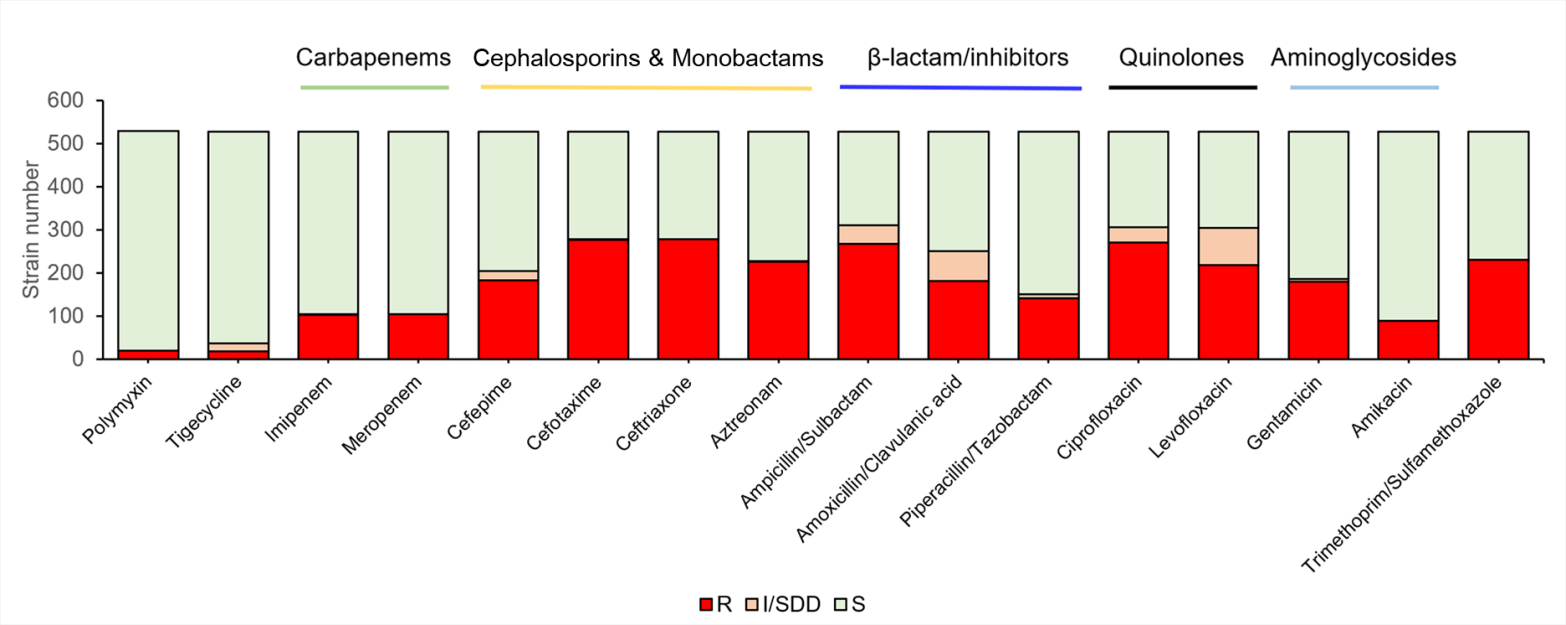
Antibiotic resistance phenotypes. R, resistant; I, intermediate; SDD, susceptible-dose dependent; S, susceptible.

Antibiotic susceptibility tests were performed for 16 antibiotics including carbapenems, cephalosporins, monobactams, β-lactam/β-lactamase combinations, quinolones, aminoglycosides, trimethoprim-sulfamethoxazole, and two last-line antibiotics, polymyxin and tigecycline (Fig. 4 and Table S3). Resistance levels were in general expected: high levels of resistance for cephalosporins and quinolones, lower levels for carbapenems, and very low levels for last-line antibiotics.

### Correlated presence of virulence factors with *K. pneumoniae* types

**Fig 5:**
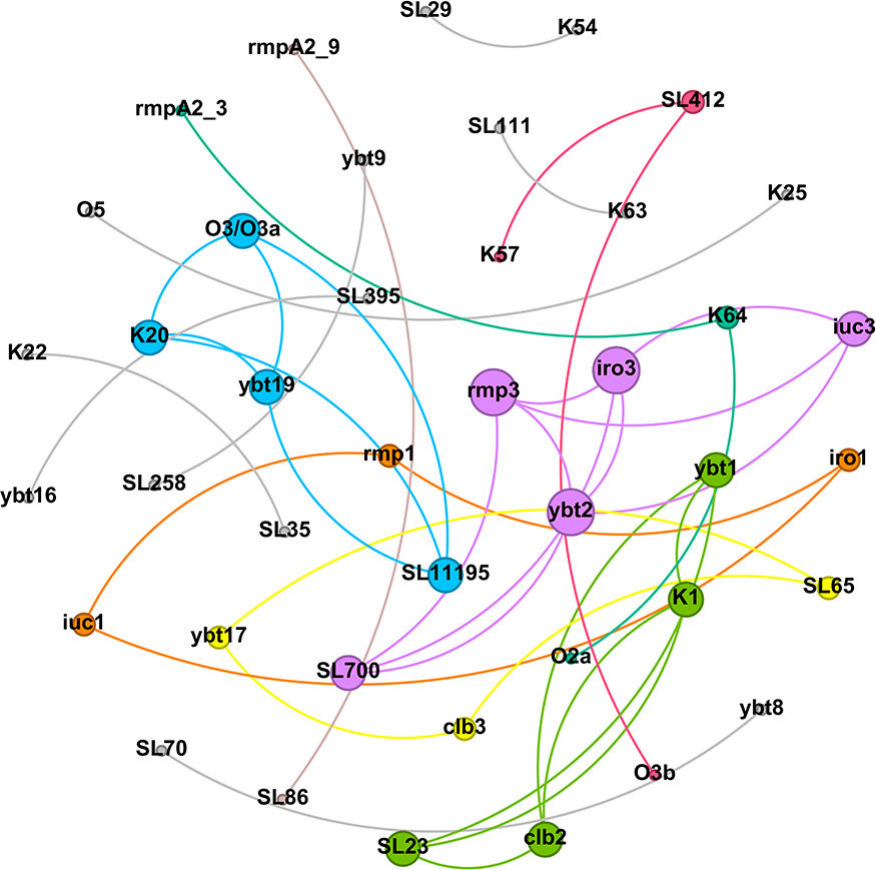
Co-occurrence network analysis of sublineages, virulence factors, and serotypes.

The correlation between sequence types, serotypes, and presence of virulence factors was calculated, leading to the generation of a co-occurrence network (Fig. 5). The virulence factors were found to be generally correlated between each other, suggesting aggregation of virulence factors (Table 1), although some correlations are significantly stronger than others. For instance, stronger correlation of *iro-rmp* (*R* = 0.9492) was found than *ybt-iuc* (*R* = 0.2896), which is a sign of preference of virulence co-occurrence. Additionally, the presence of virulence factors was compared between hypervirulent sublineages SL23, SL65, SL85, SL412 (26), and SL29 (27) and MDR sublineages SL258, SL15, SL395 (28), SL37 (29), and SL147 (30) (Table 2). Unsurprisingly, strong presence of virulence factors was found for hypervirulent sublineages.

**TABLE 1:** Correlation of the presence of virulence factors^*a*^

	*clb*	*iuc*	*iro*	*rmp*	*rmpA2*
*ybt*	0.3582	0.2896	0.2209	0.2381	0.2478
	1.59 × 10^−15^	7.26 × 10^−10^	1.38 × 10^−5^	1.57 × 10^−6^	4.24 × 10^−7^
*clb*		0.5074	0.5293	0.5088	0.5569
		9.30 × 10^−34^	2.73 × 10^−37^	5.59 × 10^−34^	4.05 × 10^−42^
*iuc*			0.6763	0.7097	0.8751
			1.90 × 10^−69^	1.49 × 10^−79^	2.87 × 10^−165^
*iro*				0.9492	0.6136
				2.31 × 10^−263^	1.31 × 10^−53^
*rmp*					0.6604
					4.61 × 10^−65^

^*a*^ On top of each cell is the Pearson *R*-value; on the bottom of each is the adjusted Pearson *P*-value.

**TABLE 2:** Presence of virulence factors in sublineages^*a*^

Sublineage	*ybt*	*clb*	*iuc*	*iro*	*rmpADC*	*rmpA2*
Hypervirulent sublineages						
SL23	98.86%	98.86%	98.86%	93.18%	93.18%	96.59%
SL65	56.25%	56.25%	100.00%	100.00%	100.00%	100.00%
SL86	69.23%	0.00%	92.31%	100.00%	100.00%	92.31%
SL412	5.26%	0.00%	94.74%	100.00%	100.00%	89.47%
SL29	58.82%	0.00%	23.53%	58.82%	58.82%	23.53%
MDR sublineages						
SL258	97.87%	1.06%	51.06%	2.13%	11.70%	47.87%
SL15	45.45%	0.00%	4.55%	0.00%	0.00%	0.00%
SL395	100.00%	0.00%	0.00%	0.00%	0.00%	0.00%
SL37	10.52%	0.00%	0.00%	0.00%	0.00%	0.00%
SL147	5.88%	0.00%	0.00%	0.00%	0.00%	0.00%

^*a*^ Gray color indicates low presence.

A general lack of virulence factors was found for MDR sublineages, except for *ybt* genes that are present in SL258, SL15, and SL395. Further analysis showed strong correlation of specific *ybt* types with sequence types. Among the 11 *ybt* types found in more than 5 strains, 7 were found associated with a specific sequence type. This finding prompts us to wonder whether the evolution of *ybt* genotypes coincides with the evolution of sequence types. Considering *ybt* is involved in immune escape, it makes sense that the selection pressure from the immune system may have played a role in the evolution of *K. pneumoniae*, during which *ybt* evolution is a key factor.

Co-occurrence network analysis found clear modules, including six multicomponent structures: (i) SL23-K1-*ybt1-clb2*, (ii) SL11195-K20-O3/O3a-*ybt19*, (iii) SL65-*ybt17-clb3*, (iv) SL700-*ybt2-iro3-iuc3-rmp3*, (v) SL412-O3b-K57, and (vi) *rmp1-iuc1-iro1*. These structures, most of which include hypervirulent sequence types, reveal that many virulent factor types are correlated with *K. pneumoniae* types, which also establishes the baseline of virulence genotype maps in important hypervirulent *K. pneumoniae* types of the region.

### Emergence of new clusters and diversification of *K. pneumoniae* community post-pandemic

**Fig 6:**
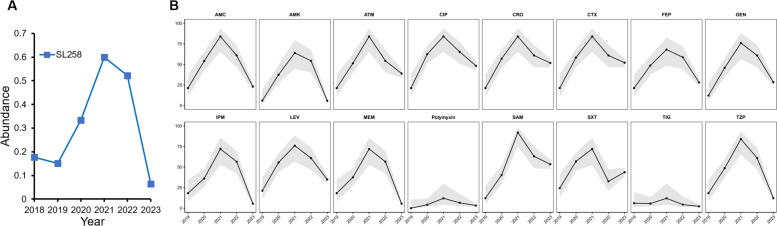
Year-to-year changes of SL258 and antibiotic resistance phenotypes. (**A**) Annual changes in the abundance of SL258 from 2018 to 2023; (**B**) year-to-year non-susceptibility rates for each antibiotic, and the shaded areas represent the 95% confidence intervals. R, Resistant; I, intermediate; SDD, susceptible-dose dependent; S, susceptible; TIG, tigecycline; IPM, imipenem; MEM, meropenem; FEP, cefepime; CTX, cefotaxime; CRO, ceftriaxone; ATM, aztreonam; SAM, ampicillin/sulbactam; AMC, amoxicillin/clavulanic acid; TZP, piperacillin/tazobactam; CIP, ciprofloxacin; LEV, levofloxacin; GEN, gentamicin; AMK, amikacin; SXT, trimethoprim/sulfamethoxazole. *Y*-axis in Panel B indicates non-susceptibility rate (R combined with I/SDD).

Year-to-year analysis found that the most frequently observed MDR *K. pneumoniae* sequence type SL258 increased starting from 2019 and began to drop in 2022, returning to pre-2019 levels in 2023 (Fig. 6A). This change coincides with the COVID-19 pandemic that started in late 2019 and ended in late 2022. Year-to-year comparison of non-susceptibility rates among all *K. pneumoniae* strains revealed a significant upward trend in resistance to multiple clinical agents (Fig. 6B and Table S4): non-susceptibility rates to the most antibiotics increased substantially during the pandemic period and declined post-pandemic, and the corresponding 95% confidence intervals showed minimal overlap with those of 2021–2022, suggesting that the decrease was unlikely to be explained solely by sampling variation. This is a distinct feature from CHINET for all reported *K. pneumoniae* strains in China (2), where such trend was only observed for ciprofloxacin. While certain antibiotics like tigecycline (*P*_trend_ = 0.388) and polymyxin (*P*_trend_ = 0.133) showed numerical increases in resistance rates, these changes did not reach statistical significance.

The change of SL258 during the COVID pandemic can partially explain the changes of antimicrobial susceptibility of the *K. pneumoniae* community. However, the frequency of SL258 dropped by eightfold (from 0.52 in 2022 to 0.06 in 2023) post-pandemic, whereas the levels of AMR only dropped by 1.4-fold. Therefore, other factors must have played a role. This led to the proposal that the diversification of MDR sequence types from SL258-dominant pre-pandemic to other sequence types may have contributed to this phenomenon.

**Fig 7:**
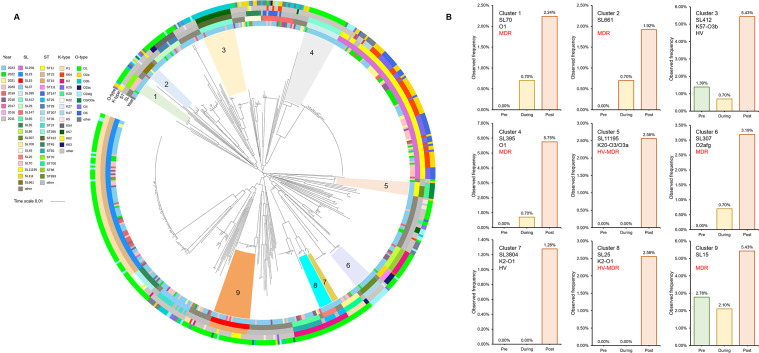
Emergence or expansion of *K. pneumoniae* clusters post-pandemic. (**A**) Phylogenetic analysis of all *K. pneumoniae* strains; (**B**) comparison of *K. pneumoniae* clusters pre-, during-, and post-pandemic. HV, hypervirulent; MDR, multidrug resistant; HV-MDR, hypervirulent-multidrug-resistant. Percentages indicate the ratio of strains to all strains collected during the time period.

Further analysis of all *K. pneumoniae* strains supports this proposal. With phylogenetic analysis, nine evolutionarily related clusters that were newly emerged or substantially expanded post-pandemic were identified (Fig. 7 and Table S5). These clusters include two newly emerged hypervirulent MDR clades (SL11195 and SL25) and five substantially expanded MDR clades (SL70, SL661, SL395, SL307, and SL15). Only two clusters are hypervirulent *K. pneumoniae* types. All of these newly emerged or substantially expanded MDR strains account for 23.64% of all *K. pneumoniae* strains post-pandemic, suggesting a strong diversification of MDR *K. pneumoniae* away from the SL258 dominance.

**Fig 8:**
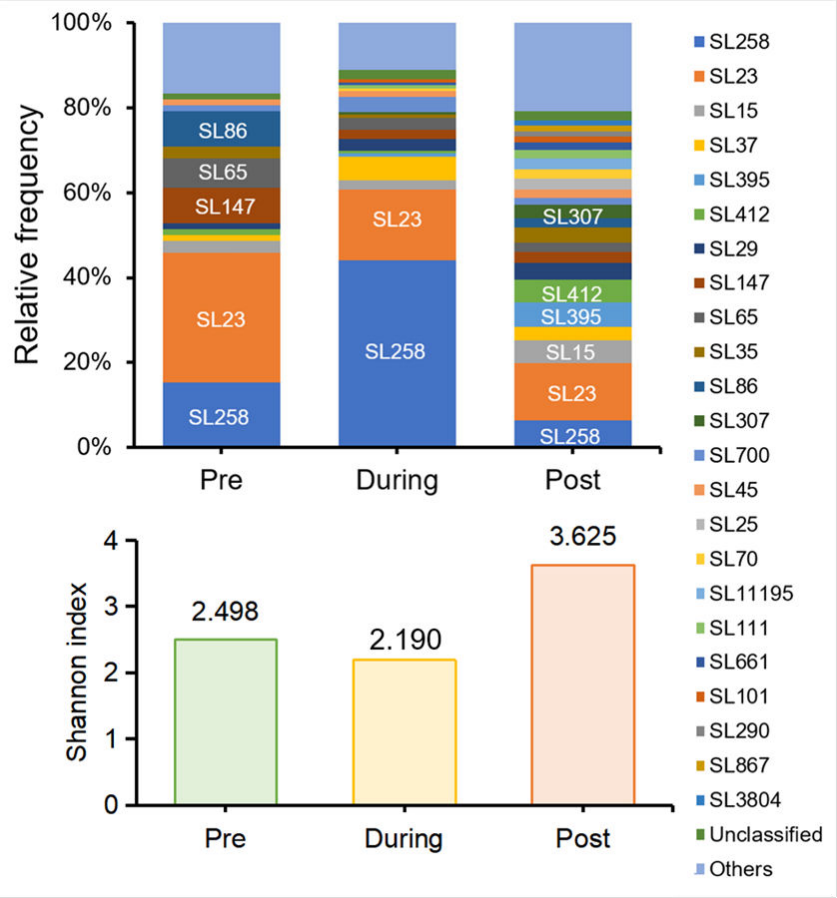
Diversification of *K. pneumoniae* types post-pandemic.

Inspection of relative frequencies of *K. pneumoniae* sequence types supports the diversification of *K. pneumoniae* strains as a community, which is not limited to MDR *K. pneumoniae* strains (Fig. 8). The *K. pneumoniae* types were generally quite simple pre-pandemic, with SL258, SL23, SL147, SL86, and SL65 as the major sequence types. The community structure was further simplified during the pandemic, with the disappearance of hypervirulent SL86 and SL65 and multidrug-resistant SL147 types, leaving SL258 and SL23 as the predominant sequence types. However, the community complexity drastically increased after the pandemic, with the appearance of many sequence types described above with phylogenetic analysis. The Shannon index was calculated based on the frequency of each SL, which supports this finding: the Shannon index dropped during-pandemic and drastically increased post-pandemic (Fig. 8). The rarefied Shannon index was also calculated to ensure that the increased diversity observed in 2023 was not a bias resulting from a larger sample size (Fig. S1). Even when normalized to a consistent sampling depth (*n* = 72), the diversity in the post-pandemic period (H′ = 3.314) remained substantially higher than that during the pandemic (H′ = 2.045).

### Correlation of AMR with *K. pneumoniae* types and replacement of SL258 during the pandemic

**Fig 9:**
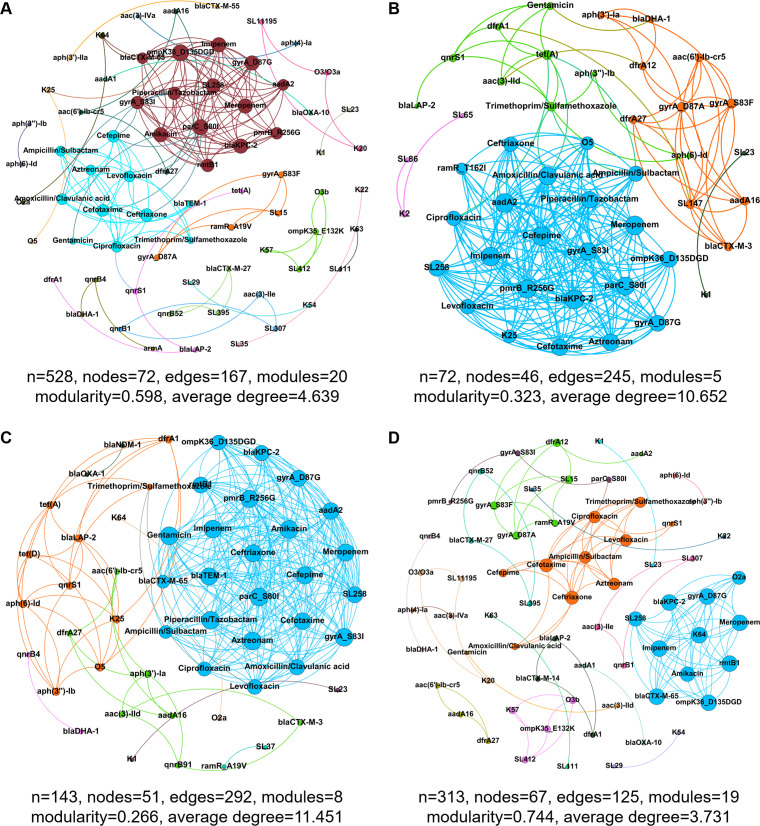
Co-occurrence network analysis of *K. pneumoniae* sequence types and AMR. (**A**) Aggregated analysis of all strains; (**B**) pre-pandemic strains; (**C**) during-pandemic strains; (**D**) post-pandemic strains.

Co-occurrence network of *K. pneumoniae* sequence, serotypes, and AMR was constructed and analyzed (Fig. 9). Dramatic changes in the networks were found pre-, during-, and post-pandemic. Most notably, modularity (the degree of each node/feature forming isolated modules with surrounding nodes) dropped during-pandemic and increased by over twofold post-pandemic. Meanwhile, the average degree (the average number of edges per node) increased during-pandemic, and dropped by 2.85-fold post-pandemic. This is a strong suggestion that modules merged during-pandemic and isolated modules emerged post-pandemic, reflecting diversification of *K. pneumoniae* types and re-balance of the community post-pandemic.

Three primary modules were present in the co-occurrence network pre-pandemic: (i) a SL258 module containing serotypes K25-O5, carbapenem and piperacillin/tazobactam resistance phenotype, antimicrobial-resistant mutations, and *bla*_KPC-2_ and *aadA2*, (ii) a SL147 module containing antimicrobial-resistant mutations and genes, and (iii) a module containing trimethoprim/sulfamethoxazole-resistant and antimicrobial-resistant mutations and genes. All three modules are still present during-pandemic, with two obvious changes: (i) the SL258 module enhanced incorporating other cephalosporin, quinolone, and gentamicin resistance; (ii) the SL147 module lost SL147, most probably because of the disappearance of SL147 during-pandemic (Fig. 8).

The co-occurrence network drastically changed post-pandemic. Of all three major modules pre- and during-pandemic, only the SL258 module remained, with two substantial changes: (i) most resistance phenotypes except for carbapenem resistance, while still correlated, lost connection with SL258 and carbapenem resistance, resulting in the split of the SL258 module; (ii) SL258 lost connection with K25 and O5 but gained connection with K64 and O2a. Also, a series of small modules appeared, in line with the increase of modularity and decrease of average degree. For instance, SL15, SL412, SL307, and SL398 modules appeared, each centered around an emerging or expanding *K. pneumoniae* cluster post-pandemic.

**Fig 10:**
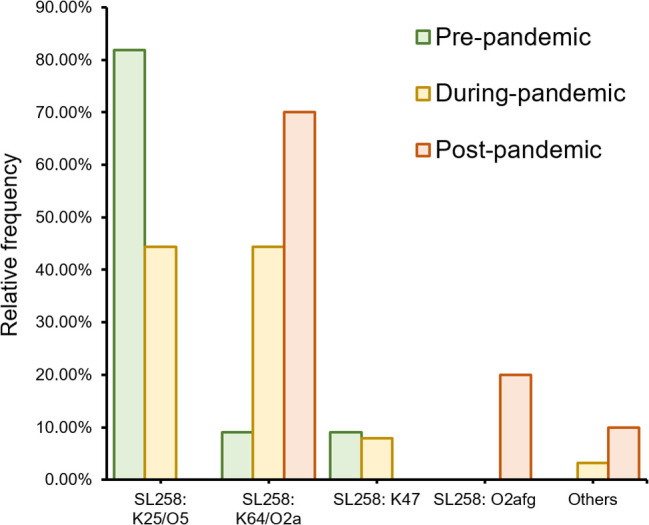
Changes in the serotypes of *K. pneumoniae* SL258 strains.

The changes of the SL258 module reflect the replacement of SL258 strains around the COVID-19 pandemic. As further shown in Fig. 10, most SL258 *K. pneumoniae* strains belonged to the K25/O5 type pre-pandemic, which gradually diminished during- and post-pandemic. Meanwhile, less than 10% of the SL258 strains were K64/O2a type pre-pandemic, but the pandemic turned it into the predominant SL258 type. Other minor types such as the O2afg type also emerged post-pandemic. This finding showed a major event happening in the *K. pneumoniae* community around the pandemic: although SL258 is still an important MDR *K. pneumoniae* type post-pandemic, strain replacement from K25/O5 to K64/O2a serotypes took place.

## DISCUSSION

The COVID-19 pandemic has exerted an unprecedented impact on global public health systems and profoundly altered the epidemiological characteristics of bacteria, particularly regarding AMR and virulence. Against this backdrop, *K. pneumoniae*, a crucial opportunistic pathogen, has also undergone significant epidemiological changes. Our comprehensive analysis of 528 clinical *K. pneumoniae* strains collected in Qingdao, China, before, during, and after the pandemic revealed a complete rebalancing of the *K. pneumoniae* community structure. This rebalancing was characterized by a simplification of strain types, with MDR SL258 becoming predominant during the pandemic. Post-pandemic, the community became more complex, marked by the emergence or expansion of numerous previously less-frequent *K. pneumoniae* types, including both MDR and hypervirulent MDR strains. Furthermore, we observed a strain replacement event within the SL258 sublineage, where K25/O5 types prevalent pre-pandemic were replaced by K64/O2a types post-pandemic. These findings collectively illustrate a profound overhaul of the *K. pneumoniae* ecosystem driven by the pandemic, offering unique insights into how societal behaviors can shape pathogen landscapes.

Our findings align with several global observations regarding the impact of the pandemic on AMR. Studies consistently indicate a marked increase in healthcare-associated AMR infections during the pandemic, with a notable rise in the transmission of MDR gram-negative bacteria, including *K. pneumoniae* (31). This phenomenon is primarily attributable to widespread empirical antibiotic use in COVID-19 patients (32), substantial increases in broad-spectrum antibiotic and carbapenem consumption (33), overwhelmed healthcare systems leading to challenges in executing infection control measures (34), and disturbances in patient immune function (35–37) and microbiome dysbiosis (38). Collectively, these factors facilitated the dissemination and clonal expansion of resistant strains. Specifically, carbapenem-resistant *K. pneumoniae* (CRKP) strains isolated during the pandemic were found to harbor pLVPK-like virulence plasmids, conferring high virulence traits (39). This observation suggests a potential co-selection between resistance and virulence characteristics, a trend also noted in other studies. In parallel, pandemic-related disruptions in infection prevention and control practices were also associated with transient increases in CRKP prevalence in ICU settings (40). However, because our study did not directly collect hospital-level antimicrobial usage or infection control data, these factors should be interpreted as potential contributing mechanisms rather than confirmed causes in our cohort.

Genomic surveillance, such as whole-genome sequencing (WGS), has unveiled the clonal expansion and nosocomial transmission of specific resistant *K. pneumoniae* strains (e.g., ST11 and ST15), with even new subclones emerging after recombination events (41). However, our study also revealed a distinct post-pandemic decline in overall AMR levels across all antibiotic types in Qingdao, a trend not uniformly observed in broader Chinese surveillance data (e.g., CHINET, where only ciprofloxacin showed such a decline) (2). These localized patterns might be attributed to local infection control strategies or the unique dynamics of *K. pneumoniae* population restructuring observed in our cohort. Moreover, our detailed analysis of community complexity, showing a simplification during and a drastic increase post-pandemic with the identification of specific emerging clusters, provides a more granular understanding of the pandemic’s influence on pathogen diversity than previously available. The observed re-seeding of previously outcompeted strain types post-pandemic, leading to a complex dynamic community, highlights the profound and long-lasting effects of large-scale societal disruptions on microbial ecosystems.

The shift in *K. pneumoniae* dynamics observed in our study is consistent with recent global reports highlighting the pandemic’s role as a catalyst for bacterial evolution. Regarding resistance and genetic diversity, our findings echo a retrospective study from a Brazilian tertiary hospital (42), which reported that carbapenem resistance in *K. pneumoniae* doubled during the pandemic. Notably, the Brazilian study observed a significant increase in genetic diversity among *bla*_KPC_-positive clones and a shift in mobile genetic elements, which parallels the diversification of sequence types and the expansion of specific MDR clusters identified in our cohort post-pandemic. In terms of the broader impact, while national data from France (43) indicated that strict infection control could temporarily mitigate ESBL-KP spread, evidence from both Brazil and Northern Ghana (44) suggests a more persistent upward trend in resistance. This divergence emphasizes that in environments with high antibiotic selection pressure—such as during the treatment of severe COVID-19—the emergence of high-risk, hypervirulent-multidrug-resistant *K. pneumoniae* strains may outpace the benefits of conventional hygiene protocols. Our identification of the SL258 cluster’s persistence further underscores this global concern of “superbug” evolution in the post-pandemic landscape.

This study benefits significantly from the integration of WGS and phenotypic data, a comprehensive approach that was previously cost-prohibitive for large-scale routine surveillance but is now increasingly feasible. The findings underscore the power of WGS as a routine clinical measure for tracking pathogen trends with unprecedented resolution. The observed dramatic changes in *K. pneumoniae* community structures, despite the re-balancing, confirm the validity and stability of baseline strain type-AMR-virulence correlation maps (Fig. 5 and 9A). For instance, the *iro1-iuc1-rmp1* and the *aadA16-aac(6’)-Ib-cr5-dfrA27* structures represent stable connections among virulence factors and ARGs that remained consistent even amidst a community structure overhaul. However, this study also has limitations. As a retrospective study of collected strains, its statistical power may be inherently lower compared to a prospective study. The annual distribution of isolates fluctuated significantly over the 6-year period: fewer than 50 isolates were collected in 2018, 2019, 2021, and 2022, respectively, with fewer than 100 in 2020. Notably, 2023 accounted for 313 isolates, representing a substantial disproportion that may introduce bias into the results and their subsequent interpretation. Nevertheless, the magnitude of the observed changes in *K. pneumoniae* community structure provides strong confidence in the validity of our results. Another limitation is the source of the strains, which were collected from hospitalized patients rather than the broader urban community. This may limit the generalizability of our findings to community-acquired infections. Besides, as a single-center study, the observed clonal shifts and resistance trends may reflect local epidemiology and clinical practices, which may not be fully representative of other geographical regions. What’s more, variations in annual sampling rates, although analyzed using proportions and trend tests, could still be influenced by changes in healthcare-seeking behavior during the COVID-19 pandemic, and the variable sampling rates across the pandemic years may affect the granularity of our observations. Future work will address this by comparing *K. pneumoniae* characteristics from community-origin and hospital-origin strains to identify their correlations, and multicenter studies are needed to validate these findings on a broader scale. Finally, the use of short-read sequencing technology limited our ability to fully characterize the structural evolution of large resistance plasmids, which warrants further investigation using long-read sequencing platforms.

In conclusion, this work provides an in-depth analysis of how the COVID-19 pandemic, acting as a full-scale disruptive event, led to a complete rebalancing of the *K. pneumoniae* community structure. This shift was primarily driven by the widespread empirical antibiotic use in hospitals, coupled with overburdened healthcare systems hindering effective infection control, and impaired patient immunity. These factors collectively promoted the spread and clonal expansion of resistant *K. pneumoniae* strains. Concurrently, social distancing and self-protective measures influenced bacterial transmission dynamics at the community level. The strict social distancing and self-protection measures undertaken during the pandemic effectively reduced *K. pneumoniae* transmission and intra-species competition within the same host, likely leading to the selection of strains better able to tolerate immune and antibiotic pressure. The removal of these measures post-pandemic restored transmission and intra-species competition, initiating a new dynamic equilibrium. This unprecedented social behavior offered a rare opportunity to observe strain population competition and dynamics being re-established after a period of near-complete isolation. Future surveillance studies are crucial to determine if the *K. pneumoniae* community structure will return to its pre-pandemic baseline, whether a permanent “scar” will remain, and how long this re-balancing process will take. Ultimately, this work offers a unique perspective on the consequences of social measures in countering a pandemic, providing valuable knowledge for better preparedness and response to future public health crises.

### Conclusion

Through genomic and phenotypic analysis of 528 *K. pneumoniae* strains from Qingdao, this study revealed the profound impact of the COVID-19 pandemic on its community structure. We found that during the pandemic, the *K. pneumoniae* community significantly simplified, dominated by MDR SL258. Post-pandemic, community complexity increased, with the emergence and expansion of various novel or expanding strains. Notably, a strain replacement occurred within the MDR SL258 sublineage, where the K64/O2a serotype largely replaced the previously dominant K25/O5 type. These dynamic changes are attributed to a complex interplay of widespread antibiotic use, healthcare system pressures, altered host immunity, and the implementation and subsequent relaxation of stringent social intervention measures. Furthermore, this study successfully mapped the relationships among strain types, AMR, and virulence features, providing an important baseline for *K. pneumoniae* community characteristics in the region. These findings underscore the critical role of large-scale societal interventions in shaping pathogen evolution and highlight the necessity for continuous genomic surveillance to inform future public health strategies.

## Data Availability

All whole genome sequences are deposited in GenBank under Bioproject no. PRJNA1205678.

## SUPPLEMENTAL MATERIAL

Fig. S1 aem.00706-26-s0001.tif Rarefied Shannon diversity indices of *K. pneumoniae* types across different pandemic periods.

Supplemental material aem.00706-26-s0002.docx Fig. S1 legend.

Table S1 aem.00706-26-s0003.xlsx *K. pneumoniae* strains studied in this work.

Table S2 aem.00706-26-s0004.xlsx Carriage of antibiotic resistance genes or mutations.

Table S3 aem.00706-26-s0005.xlsx Antibiotic susceptibility phenotypes of isolated strains.

Table S4 aem.00706-26-s0006.xlsx Statistical analysis of antibiotic resistance trends during the COVID-19 pandemic (2019-2022).

Table S5 aem.00706-26-s0007.docx Emerged or expanded *K. pneumoniae* clusters post-pandemic.

ASM does not own the copyrights to Supplemental Material that may be linked to, or accessed through, an article. The authors have granted ASM a non-exclusive, world-wide license to publish the Supplemental Material files. Please contact the corresponding author directly for reuse.
