# Supplementary figures and images for: Dynamics of clinical Klebsiella pneumoniae strains over the COVID-19 pandemic in Qingdao, China
Source: Appl Environ Microbiol. 2026 Jun 29;92(7):e00706-26. doi: 10.1128/aem.00706-26 (PMC13390487; doi:10.1128/aem.00706-26)

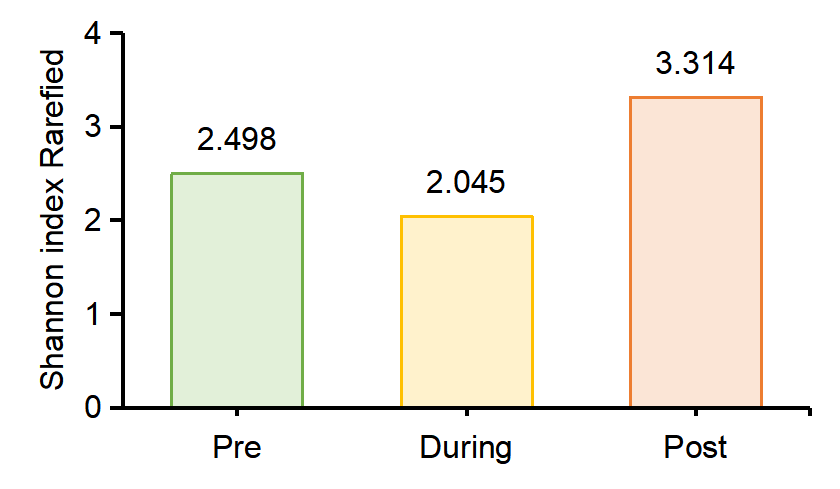

Supplement: Fig. S1 — Rarefied Shannon diversity indices of K. pneumoniae types across different pandemic periods. [file aem.00706-26-s0001.tif]
