# Supplementary material for: Dynamics of clinical Klebsiella pneumoniae strains over the COVID-19 pandemic in Qingdao, China
Source: Appl Environ Microbiol. 2026 Jun 29;92(7):e00706-26. doi: 10.1128/aem.00706-26 (PMC13390487; doi:10.1128/aem.00706-26)
Supplement: Supplemental material — Fig. S1 legend. [file aem.00706-26-s0002.docx]

Figure S1. Rarefied Shannon diversity indices of K. pneumoniae types across different pandemic periods.
