# Supplementary material for: Dynamics of clinical Klebsiella pneumoniae strains over the COVID-19 pandemic in Qingdao, China
Source: Appl Environ Microbiol. 2026 Jun 29;92(7):e00706-26. doi: 10.1128/aem.00706-26 (PMC13390487; doi:10.1128/aem.00706-26)
Supplement: Table S5 — Emerged or expanded K. pneumoniae clusters post-pandemic. [file aem.00706-26-s0007.docx]

**Table S5. Emerged or expanded *K. pneumoniae* clusters post-pandemic.**

| **Strains** | **AMR phenotype** | **ARGs** | **virulence factors** |
| --- | --- | --- | --- |
| SL70 | | | |
| C1-14 | Ampicillin/Sulbactam, Amoxicillin/Clavulanic acid, Piperacillin/Tazobactam | *tet*(D) | NA |
| E3-13 | Cefotaxime, Ceftriaxone, Aztreonam, Ampicillin/Sulbactam, Ciprofloxacin, Levofloxacin | *bla*_CTX-M-15_, *qnrS1* | *ybt8* |
| C6-80 | Cefepime, Cefotaxime, Ceftriaxone, Aztreonam, Ampicillin/Sulbactam, Amoxicillin/Clavulanic acid, Piperacillin/Tazobactam, Ciprofloxacin, Levofloxacin | *bla*_CTX-M-15_, *qnrS1* | *ybt8* |
| C6-50 | Cefepime, Cefotaxime, Ceftriaxone, Aztreonam, Ampicillin/Sulbactam, Amoxicillin/Clavulanic acid, Ciprofloxacin, Levofloxacin | *bla*_CTX-M-15_, *qnrS1* | *ybt8* |
| C5-13 | Cefotaxime, Ceftriaxone, Aztreonam, Ampicillin/Sulbactam, Amoxicillin/Clavulanic acid, Ciprofloxacin, Levofloxacin | *bla*_CTX-M-15_, *qnrS1* | *ybt8* |
| C4-7 | Cefotaxime, Ceftriaxone, Ciprofloxacin, Levofloxacin | *bla*_CTX-M-15_, *qnrS1* | *ybt8* |
| C4-11 | Cefotaxime, Ceftriaxone, Ciprofloxacin, Levofloxacin | *bla*_CTX-M-15_, *qnrS1* | *ybt8* |
| C1-177 | Cefotaxime, Ceftriaxone, Aztreonam, Ciprofloxacin, Levofloxacin | *bla*_CTX-M-15_, *qnrS1* | *ybt8* |
| SL661 | | | |
| C6-32 | Cefotaxime, Ceftriaxone, Ampicillin/Sulbactam, Amoxicillin/Clavulanic acid, Ciprofloxacin, Levofloxacin, Gentamicin | *aac(3)-IId*, *bla*_CTX-M-14_, *bla*_LAP-2_, *qnrS1*, *tet*(A), *dfrA1* | NA |
| C6-82 | Cefotaxime, Ceftriaxone, Ampicillin/Sulbactam, Ciprofloxacin, Levofloxacin | *aph(6)-Id*, *aph(3’’)-Ib*, *bla*_TEM-1_, *bla*_CTX-M-3_, *qnrS1*, *tet*(A) | *iuc3* |
| C6-115 | Cefotaxime, Ceftriaxone, Ampicillin/Sulbactam, Ciprofloxacin, Levofloxacin | *qnrS1*, *tet*(D), *dfrA14* | NA |
| C1-4 | Ampicillin/Sulbactam, Amoxicillin/Clavulanic acid, Piperacillin/Tazobactam | *tet*(D) | NA |
| E3-2 | Cefepime, Cefotaxime, Ceftriaxone, Aztreonam, Ampicillin/Sulbactam, Ciprofloxacin, Levofloxacin, Trimethoprim/Sulfamethoxazole | *aph(6)-Id*, *aph(3'')-Ib*, *bla*_TEM-1_, *bla*_CTX-M-3_, *qnrS1*, *tet*(A) | NA |
| C6-39 | Cefotaxime, Ceftriaxone, Aztreonam, Ampicillin/Sulbactam, Amoxicillin/Clavulanic acid, Ciprofloxacin, Levofloxacin, Trimethoprim/Sulfamethoxazole | *aph(6)-Id*, *aph(3'')-Ib*, *bla*_TEM-1_, *bla*_CTX-M-15_, *qnrB1*, *tet*(A), *dfrA14* | NA |
| C6-133 | Cefotaxime, Ceftriaxone, Aztreonam, Ampicillin/Sulbactam, Amoxicillin/Clavulanic acid, Ciprofloxacin, Levofloxacin | *aph(3')-Ia*, *bla*_DHA-1_, *bla*_TEM-1_, *bla*_LAP-2_, *bla*_CTX-M-3_, *qnrS1*, *qnrB4* | NA |
| SL412 | | | |
| C4-2 | NA | *ompK35_E132K* | *iuc1*, *iro1* |
| C1-40 | NA | *ompK35_E132K* | *iuc1*, *iro1* |
| C6-45 | NA | *ompK35_E132K* | *iro1* |
| C1-121 | Polymyxin | *ompK35_E132K*, *tet*(C) | *iuc1*, *iro1* |
| C1-111 | NA | *ompK35_E132K*, *tet*(C) | *iuc1*, *iro1* |
| C1-151 | NA | *ompK35_E132K* | *iuc1*, *iro1* |
| C1-167 | NA | *ompK35_E132K* | *ybt9*,  *iuc1*, *iro1* |
| C1-148 | NA | *ompK35_E132K*, *tet*(C) | *iuc1*, *iro1* |
| C6-51 | NA | *ompK35_E132K* | *iuc1*, *iro1* |
| E3-42 | NA | *ompK35_E132K* | *iuc1*, *iro1* |
| C6-33 | NA | *ompK35_E132K* | *iuc1*, *iro1* |
| C5-32 | NA | *ompK35_E132K* | *iuc1*, *iro1* |
| C5-16 | NA | *ompK35_E132K* | *iuc1*, *iro1* |
| C4-16 | NA | *ompK35_E132K* | *iuc1*, *iro1* |
| C1-94 | Ampicillin/Sulbactam | *ompK35_E132K*, *tet*(C) | *iuc1*, *iro1* |
| C1-175 | Polymyxin | *ompK35_E132K* | *iuc1*, *iro1* |
| C1-163 | NA | *ompK35_E132K* | *iuc1*, *iro1* |
| C1-149 | NA | *ompK35_E132K* | *iuc1*, *iro1* |
| C1-125 | Polymyxin | *ompK35_E132K*, *tet*(C) | *iuc1*, *iro1* |
| SL395 | | | |
| C1-113 | Cefepime, Cefotaxime, Ceftriaxone, Aztreonam, Ampicillin/Sulbactam, Amoxicillin/Clavulanic acid, Ciprofloxacin, Levofloxacin, Trimethoprim/Sulfamethoxazole | *aadA16*, *aac(3)-IId*, *bla*_CTX-M-27_, *pmrB_R256G*, *parC_S80I*, *aac(6')-Ib-cr5*, *gyrA_S83I*, *qnrB52*, *tet*(C), *dfrA27* | *ybt16* |
| C1-124 | Cefotaxime, Ceftriaxone, Aztreonam, Ampicillin/Sulbactam, Amoxicillin/Clavulanic acid, Piperacillin/Tazobactam, Ciprofloxacin, Levofloxacin, Trimethoprim/Sulfamethoxazole | *aadA16*, *bla*_CTX-M-27_, *bla*_OXA-1_, *pmrB_R256G*, *parC_S80I*, *aac(6')-Ib-cr5*, *gyrA_S83I*, *qnrB52*, *tet*(C), *tet*(A), *dfrA27* | *ybt16* |
| C4-12 | Cefepime, Cefotaxime, Ceftriaxone, Aztreonam, Ampicillin/Sulbactam, Amoxicillin/Clavulanic acid, Ciprofloxacin, Levofloxacin, Trimethoprim/Sulfamethoxazole | *aadA16*, *bla*_CTX-M-27_, *pmrB_R256G*, *parC_S80I*, *aac(6')-Ib-cr5*, *gyrA_S83I*, *qnrB52*, *dfrA27* | *ybt16* |
| C4-19 | Cefepime, Cefotaxime, Ceftriaxone, Aztreonam, Ampicillin/Sulbactam, Ciprofloxacin, Levofloxacin, Trimethoprim/Sulfamethoxazole | *aadA16*, *bla*_CTX-M-27_, *pmrB_R256G*, *parC_S80I*, *aac(6')-Ib-cr5*, *gyrA_S83I*, *qnrB52*, *dfrA27* | *ybt16* |
| C4-35 | Cefepime, Cefotaxime, Ceftriaxone, Aztreonam, Ampicillin/Sulbactam, Ciprofloxacin, Levofloxacin, Trimethoprim/Sulfamethoxazole | *aadA16*, *bla*_CTX-M-27_, *pmrB_R256G*, *parC_S80I*, *aac(6')-Ib-cr5*, *gyrA_S83I*, *qnrB52*, *dfrA27* | *ybt16* |
| C4-36 | Levofloxacin | *aadA16*, *bla*_CTX-M-27_, *pmrB_R256G*, *parC_S80I*, *aac(6')-Ib-cr5*, *gyrA_S83I*, *qnrB52*, *tet(C)*, *dfrA27* | *ybt16* |
| C5-11 | Cefepime, Cefotaxime, Ceftriaxone, Aztreonam, Ampicillin/Sulbactam, Amoxicillin/Clavulanic acid, Ciprofloxacin, Levofloxacin, Trimethoprim/Sulfamethoxazole, Polymyxin | *aadA16*, *bla*_CTX-M-27_, *pmrB_R256G*, *parC_S80I*, *aac(6')-Ib-cr5*, *gyrA_S83I*, *qnrB52*, *dfrA27* | *ybt16* |
| C6-101 | Cefepime, Cefotaxime, Ceftriaxone, Aztreonam, Ampicillin/Sulbactam, Ciprofloxacin, Levofloxacin, Trimethoprim/Sulfamethoxazole | *aadA16*, *bla*_CTX-M-27_, *pmrB_R256G*, *parC_S80I*, *aac(6')-Ib-cr5*, *gyrA_S83I*, *qnrB52*, *dfrA27* | *ybt16* |
| C6-11 | Cefepime, Cefotaxime, Ceftriaxone, Aztreonam, Ampicillin/Sulbactam, Amoxicillin/Clavulanic acid, Ciprofloxacin, Levofloxacin, Trimethoprim/Sulfamethoxazole | *aadA16*, *bla*_CTX-M-27_, *pmrB_R256G*, *parC_S80I*, *aac(6')-Ib-cr5*, *gyrA_S83I*, *qnrB52*, *dfrA27* | *ybt16* |
| C6-2 | Cefepime, Cefotaxime, Ceftriaxone, Aztreonam, Ampicillin/Sulbactam, Amoxicillin/Clavulanic acid, Ciprofloxacin, Levofloxacin, Trimethoprim/Sulfamethoxazole | *aadA16*, *bla*_CTX-M-27_, *pmrB_R256G*, *parC_S80I*, *aac(6')-Ib-cr5*, *gyrA_S83I*, *qnrB52*, *dfrA27* | *ybt16* |
| C6-36 | Cefepime, Cefotaxime, Ceftriaxone, Aztreonam, Ampicillin/Sulbactam, Amoxicillin/Clavulanic acid, Ciprofloxacin, Levofloxacin, Trimethoprim/Sulfamethoxazole | *aadA16*, *bla*_CTX-M-27_, *pmrB_R256G*, *parC_S80I*, *aac(6')-Ib-cr5*, *gyrA_S83I*, *qnrB52*, *dfrA27* | *ybt16* |
| C6-44 | Cefepime, Cefotaxime, Ceftriaxone, Aztreonam, Ampicillin/Sulbactam, Amoxicillin/Clavulanic acid, Ciprofloxacin, Levofloxacin, Trimethoprim/Sulfamethoxazole | *aadA16*, *bla*_CTX-M-27_, *pmrB_R256G*, *parC_S80I*, *aac(6')-Ib-cr5*, *gyrA_S83I*, *qnrB52*, *dfrA27* | *ybt16* |
| C6-53 | Cefepime, Cefotaxime, Ceftriaxone, Aztreonam, Ampicillin/Sulbactam, Ciprofloxacin, Levofloxacin, Trimethoprim/Sulfamethoxazole | *aadA16*, *bla*_CTX-M-27_, *pmrB_R256G*, *parC_S80I*, *aac(6')-Ib-cr5*, *gyrA_S83I*, *qnrB52*, *dfrA27* | *ybt16* |
| C6-71 | Cefepime, Cefotaxime, Ceftriaxone, Aztreonam, Ampicillin/Sulbactam, Ciprofloxacin, Levofloxacin, Trimethoprim/Sulfamethoxazole | *aadA16*, *bla*_CTX-M-27_, *pmrB_R256G*, *parC_S80I*, *aac(6')-Ib-cr5*, *gyrA_S83I*, *qnrB52*, *dfrA27* | *ybt16* |
| C6-81 | Cefepime, Cefotaxime, Ceftriaxone, Aztreonam, Ampicillin/Sulbactam, Ciprofloxacin, Levofloxacin, Trimethoprim/Sulfamethoxazole | *aadA16*, *bla*_CTX-M-27_, *pmrB_R256G*, *parC_S80I*, *aac(6')-Ib-cr5*, *gyrA_S83I*, *qnrB52*, *dfrA27* | *ybt16* |
| C6-85 | Cefepime, Cefotaxime, Ceftriaxone, Aztreonam, Ampicillin/Sulbactam, Ciprofloxacin, Levofloxacin, Trimethoprim/Sulfamethoxazole | *aadA16*, *bla*_CTX-M-27_, *pmrB_R256G*, *parC_S80I*, *aac(6')-Ib-cr5*, *gyrA_S83I*, *qnrB52*, *dfrA27* | *ybt16* |
| C6-86 | Cefepime, Cefotaxime, Ceftriaxone, Aztreonam, Ampicillin/Sulbactam, Amoxicillin/Clavulanic acid, Ciprofloxacin, Levofloxacin, Trimethoprim/Sulfamethoxazole | *aadA16*, *bla*_CTX-M-27_, *pmrB_R256G*, *parC_S80I*, *aac(6')-Ib-cr5*, *gyrA_S83I*, *qnrB52*, *dfrA27* | *ybt16* |
| C6-92 | Cefepime, Cefotaxime, Ceftriaxone, Aztreonam, Ampicillin/Sulbactam, Ciprofloxacin, Levofloxacin, Trimethoprim/Sulfamethoxazole | *aadA16*, *bla*_CTX-M-27_, *pmrB_R256G*, *parC_S80I*, *aac(6')-Ib-cr5*, *gyrA_S83I*, *qnrB52*, *dfrA27* | *ybt16* |
| E1-85 | Meropenem, Cefepime, Cefotaxime, Ceftriaxone, Ciprofloxacin, Levofloxacin, Gentamicin | *aadA16*, *aph(6)-Id*, *aph(3'')-Ib*, *aac(3)-IIe*, *bla*_KPC-2_, *bla*_CTX-M-27_, *bla*_TEM-1_, *pmrB_R256G*, *parC_S80I*, *aac(6')-Ib-cr5*, *gyrA_S83I*, *qnrB52*, *dfrA27*, *dfrA14* | *ybt16* |
| SL11195 | | | |
| C4-17 | NA | NA | *ybt19*, *iuc1*, *iro1* |
| C4-29 | NA | NA | *ybt19*, *iuc1*, *iro1* |
| C5-21 | Ampicillin/Sulbactam, Amoxicillin/Clavulanic acid | NA | *ybt19*, *iuc1*, *iro1* |
| C5-49 | NA | NA | *ybt19*, *iuc1*, *iro1* |
| C6-105 | Cefotaxime, Ceftriaxone, Ampicillin/Sulbactam, Amoxicillin/Clavulanic acid, Gentamicin | *aac(3)-IId*, *bla*_TEM-1_, *bla*_CTX-M-3_ | *ybt19*, *iuc1*, *iro1* |
| C6-106 | Cefotaxime, Ceftriaxone, Ampicillin/Sulbactam, Amoxicillin/Clavulanic acid, Gentamicin | *aac(3)-IId*, *bla*_TEM-1_, *bla*_CTX-M-3_ | *ybt19*, *iuc1*, *iro1* |
| C6-29 | NA | NA | *ybt19*, *iuc1*, *iro1* |
| C6-67 | Cefotaxime, Ceftriaxone, Ampicillin/Sulbactam, Amoxicillin/Clavulanic acid, Gentamicin | *aac(3)-IId*, *bla*_TEM-1_, *bla*_CTX-M-3_ | *ybt19*, *iuc1*, *iro1* |
| SL307 | | | |
| C1-138 | Cefotaxime, Ceftriaxone, Aztreonam, Ampicillin/Sulbactam, Amoxicillin/Clavulanic acid, Ciprofloxacin, Levofloxacin, Trimethoprim/Sulfamethoxazole | *aph(6)-Id*, *aph(3'')-Ib*, *bla*_TEM-1_, *bla*_CTX-M-15_, *bla*_OXA-1_, *parC_S80I, aac(6')-Ib-cr5, gyrA_S83I*, *qnrB1*, *tet*(C), *tet*(A), *dfrA14* | *ybt10* |
| C1-90 | Imipenem, Meropenem, Cefepime, Cefotaxime, Ceftriaxone, Aztreonam, Ampicillin/Sulbactam, Amoxicillin/Clavulanic acid, Piperacillin/Tazobactam, Ciprofloxacin, Levofloxacin, Gentamicin, Tigecycline, Trimethoprim/Sulfamethoxazole | *aph(6)-Id*, *aph(3'')-Ib*, *aadA1*, *aac(3)-IIe*, *bla*_TEM-1_, *bla*_NDM-1_, *bla*_CTX-M-15_, *bla*_OXA-1_, *bla*_CTX-M_, *parC_S80I*, *aac(6')-Ib-cr5*, *qnrS1*, *gyrA_S83I*, *qnrB1*, *tet*(C), *tet*(A), *dfrA14* | NA |
| C4-24 | Cefepime, Cefotaxime, Ceftriaxone, Aztreonam, Ampicillin/Sulbactam, Amoxicillin/Clavulanic acid, Piperacillin/Tazobactam, Ciprofloxacin, Levofloxacin, Trimethoprim/Sulfamethoxazole | *aph(6)-Id*, *aph(3'')-Ib*, *aph(4)-Ia*, *aac(3)-IVa*, *bla*_TEM-1_, *bla*_CTX-M-15_, *bla*_OXA-1_, *parC_S80I*, *aac(6')-Ib-cr5*, *gyrA_S83I*, *dfrA14* | NA |
| C4-25 | Cefepime, Cefotaxime, Ceftriaxone, Ampicillin/Sulbactam, Amoxicillin/Clavulanic acid, Piperacillin/Tazobactam, Ciprofloxacin, Levofloxacin, Gentamicin | *aph(6)-Id*, *aph(3'')-Ib*, *aac(3)-IIe*, *bla*_TEM-1_, *bla*_CTX-M-15_, *bla*_OXA-1_, *parC_S80I*, *aac(6')-Ib-cr5*, *gyrA_S83I*, *qnrB1*, *tet*(C), *tet*(A), *dfrA14* | NA |
| C6-10 | Cefepime, Cefotaxime, Ceftriaxone, Aztreonam, Ampicillin/Sulbactam, Amoxicillin/Clavulanic acid, Piperacillin/Tazobactam, Ciprofloxacin, Levofloxacin, Gentamicin, Trimethoprim/Sulfamethoxazole | *aadA2*, *aph(3')-Ia*, *aph(6)-Id*, *aph(3'')-Ib*, *aac(3)-IIe*, *bla*_TEM-1_, *bla*_CTX-M-15_, *bla*_OXA-1_, *parC_S80I*, *aac(6')-Ib-cr5*, *qnrS1*, *gyrA_S83I*, *qnrB1*, *tet*(A), *dfrA14* | NA |
| C6-102 | Cefepime, Cefotaxime, Ceftriaxone, Aztreonam, Ampicillin/Sulbactam, Amoxicillin/Clavulanic acid, Ciprofloxacin, Levofloxacin, Trimethoprim/Sulfamethoxazole | *aph(6)-Id*, *aph(3'')-Ib*, *bla*_TEM-1_, *bla*_CTX-M-15_, *bla*_OXA-1_, *parC_S80I*, *aac(6')-Ib-cr5*, *gyrA_S83I*, *qnrB1*, *tet*(A), *dfrA14* | *ybt10* |
| C6-127 | Cefotaxime, Ceftriaxone, Aztreonam, Ampicillin/Sulbactam, Amoxicillin/Clavulanic acid, Ciprofloxacin, Levofloxacin, Gentamicin, Trimethoprim/Sulfamethoxazole | *aph(6)-Id*, *aph(3'')-Ib*, *aac(3)-IIe*, *bla*_TEM-1_, *bla*_CTX-M-15_, *bla*_OXA-1_, *parC_S80I*, *aac(6')-Ib-cr5*, *gyrA_S83I*, *qnrB1*, *dfrA14* | NA |
| C6-47 | Cefotaxime, Ceftriaxone, Aztreonam, Ampicillin/Sulbactam, Amoxicillin/Clavulanic acid, Ciprofloxacin, Levofloxacin, Gentamicin, Trimethoprim/Sulfamethoxazole | *aph(6)-Id*, *aph(3'')-Ib*, *aac(3)-IIe*, *bla*_TEM-1_, *bla*_CTX-M-15_, *bla*_OXA-1_, *parC_S80I*, *aac(6')-Ib-cr5, gyrA_S83I*, *qnrB1*, *tet*(A), *dfrA14* | NA |
| C6-79 | Cefepime, Cefotaxime, Ceftriaxone, Aztreonam, Ampicillin/Sulbactam, Amoxicillin/Clavulanic acid, Piperacillin/Tazobactam, Ciprofloxacin, Levofloxacin, Gentamicin, Trimethoprim/Sulfamethoxazole | *aph(6)-Id*, *aph(3'')-Ib*, *aadA1*, *aac(3)-IIe*, *bla*_TEM-1_, *bla*_CTX-M-15_, *bla*_OXA-1_, *parC_S80I*, *aac(6')-Ib-cr5*, *gyrA_S83I*, *qnrB1*, *dfrA14* | NA |
| C6-98 | Cefepime, Cefotaxime, Ceftriaxone, Aztreonam, Ampicillin/Sulbactam, Amoxicillin/Clavulanic acid, Ciprofloxacin, Levofloxacin, Gentamicin, Trimethoprim/Sulfamethoxazole | *aadA16*, *aph(6)-Id*, *aph(3'')-Ib*, *aac(3)-IIe*, *bla*_TEM-1_, *bla*_CTX-M-15_, *bla*_OXA-1_, *parC_S80I*, *aac(6')-Ib-cr5*, *gyrA_S83I*, *qnrB1*, *tet*(A), *tet*(D), *dfrA27*, *dfrA14* | NA |
| E3-41 | Cefepime, Cefotaxime, Ceftriaxone, Aztreonam, Ampicillin/Sulbactam, Amoxicillin/Clavulanic acid, Piperacillin/Tazobactam, Ciprofloxacin, Levofloxacin, Gentamicin, Amikacin, Trimethoprim/Sulfamethoxazole | *aph(6)-Id*, *aph(3'')-Ib*, *aadA1*, *aac(3)-IIe*, *bla*_TEM-1_, *bla*_CTX-M-15_, *bla*_OXA-1_, *parC_S80I*, *aac(6')-Ib-cr5*, *gyrA_S83I*, *qnrB1*, *tet*(A), *dfrA14* | NA |
| SL3804 | | | |
| C4-39 | Trimethoprim/Sulfamethoxazole | *tet*(C) | *iuc1*, *iro1* |
| C5-25 | NA | NA | *iro1* |
| C5-26 | NA | NA | *iro1* |
| C6-8 | NA | NA | *iuc1*, *iro1* |
| SL25 | | | |
| C5-10 | Cefepime, Cefotaxime, Ceftriaxone, Aztreonam, Ampicillin/Sulbactam, Amoxicillin/Clavulanic acid, Ciprofloxacin, Levofloxacin, Gentamicin, Trimethoprim/Sulfamethoxazole | *aadA16*, *aac(3)-IId*, *aph(3')-Ia*, *aph(6)-Id*, *aph(3'')-Ib*, *aadA1*, *bla*_TEM-1_, *bla*_TEM-1_, *bla*_CTX-M-3_, *bla*_OXA-10_, *aac(6')-Ib-cr5*, *qnrS1*, *tet*(A), *dfrA27*, *dfrA14* | *ybt2*, *iuc3*, *iro3* |
| C5-24 | Cefepime, Cefotaxime, Ceftriaxone, Aztreonam, Ampicillin/Sulbactam, Amoxicillin/Clavulanic acid, Ciprofloxacin, Levofloxacin, Gentamicin, Trimethoprim/Sulfamethoxazole | *aadA16*, *aac(3)-IId*, *aph(3')-Ia*, *aph(6)-Id*, *aph(3'')-Ib*, *aadA1*, *bla*_TEM-1_, *bla*_TEM-1_, *bla*_CTX-M-3_, *bla*_OXA-10_, *aac(6')-Ib-cr5*, *qnrS1*, *tet(A)*, *dfrA27*, *dfrA14* | *ybt2*, *iuc3*, *iro3* |
| C5-39 | Cefepime, Cefotaxime, Ceftriaxone, Aztreonam, Ampicillin/Sulbactam, Amoxicillin/Clavulanic acid, Ciprofloxacin, Levofloxacin, Gentamicin, Trimethoprim/Sulfamethoxazole | *aac(3)-IId*, *aph(6)-Id*, *aph(3'')-Ib*, *aph(3')-IIa*, *bla*_TEM_, *bla*_CTX-M-14_, *bla*_CTX-M-55_, *qnrS1*, *tet*(A), *tet*(M), *dfrA1* | *ybt9* |
| C5-41 | Cefotaxime, Ceftriaxone, Aztreonam, Ampicillin/Sulbactam, Amoxicillin/Clavulanic acid, Ciprofloxacin, Levofloxacin, Gentamicin, Trimethoprim/Sulfamethoxazole | *aac(3)-IId*, *aph(6)-Id*, *aph(3'')-Ib*, *aph(3')-IIa*, *bla*_TEM_, *bla*_CTX-M-14_, *bla*_CTX-M-55_, *qnrS1*, *tet*(A), *tet*(M), *dfrA1* | *ybt9* |
| C6-40 | Cefepime, Cefotaxime, Ceftriaxone, Aztreonam, Ampicillin/Sulbactam, Amoxicillin/Clavulanic acid, Ciprofloxacin, Levofloxacin, Gentamicin, Trimethoprim/Sulfamethoxazole | *aadA16*, *aac(3)-IId*, *aph(3')-Ia*, *aph(6)-Id*, *aph(3'')-Ib*, *aadA1*, *bla*_TEM-1_, *bla*_TEM-1_, *bla*_CTX-M-3_, *bla*_OXA-10_, *aac(6')-Ib-cr5*, *qnrS1*, *tet*(A), *dfrA27*, *dfrA14* | *ybt2*, *iuc3*, *iro3* |
| C6-41 | Cefepime, Cefotaxime, Ceftriaxone, Aztreonam, Ampicillin/Sulbactam, Amoxicillin/Clavulanic acid, Ciprofloxacin, Levofloxacin, Gentamicin, Trimethoprim/Sulfamethoxazole | *aadA16*, *aac(3)-IId*, *aph(3')-Ia*, *aph(6)-Id*, *aph(3'')-Ib*, *aadA1*, *bla*_TEM-1_, *bla*_TEM-1_, *bla*_CTX-M-3_, *bla*_OXA-10_, *aac(6')-Ib-cr5*, *qnrS1*, *tet*(A), *dfrA27*, *dfrA14* | *ybt2*, *iuc3*, *iro3* |
| C6-5 | Ciprofloxacin, Levofloxacin | *bla*_TEM-1_, *qnrS1* | *ybt2*, *iuc3*, *iro3* |
| C6-54 | Cefotaxime, Ceftriaxone, Aztreonam, Ampicillin/Sulbactam, Ciprofloxacin, Levofloxacin, Gentamicin, Trimethoprim/Sulfamethoxazole | *aadA16*, *aac(3)-IId*, *aph(3')-Ia*, *aph(6)-Id*, *aph(3'')-Ib*, *bla*_TEM-1_, *bla*_CTX-M-3_, *aac(6')-Ib-cr5*, *qnrS1*, *tet*(A), *dfrA27* | *ybt2*, *iuc3*, *iro3* |
| SL15 | | | |
| C1-11 | Cefepime, Cefotaxime, Ceftriaxone, Aztreonam, Ampicillin/Sulbactam, Amoxicillin/Clavulanic acid, Ciprofloxacin, Levofloxacin, Trimethoprim/Sulfamethoxazole | *aadA16*, *aph(3')-Ia*, *bla*_DHA-1_, *bla*_CTX-M-27_, *parC_S80I*, *aac(6')-Ib-cr5*, *qnrB4*, *gyrA_D87A*, *gyrA_S83F*, *ramR_A19V*, *tet*(D), *dfrA27* | *ybt10* |
| C1-164 | Cefepime, Cefotaxime, Ceftriaxone, Aztreonam, Ampicillin/Sulbactam, Ciprofloxacin, Levofloxacin, Trimethoprim/Sulfamethoxazole | *aadA16*, *aadA2*, *aph(3')-Ia*, *bla*_TEM_, *bla*_CTX-M-15_, *parC_S80I*, *aac(6')-Ib-cr5*, *gyrA_D87A*, *qnrB52*, *gyrA_S83F*, *tet*(A), *ramR_A19V*, *dfrA27*, *dfrA12* | NA |
| C1-171 | Cefotaxime, Ceftriaxone, Aztreonam, Ampicillin/Sulbactam, Amoxicillin/Clavulanic acid, Ciprofloxacin, Levofloxacin, Gentamicin, Trimethoprim/Sulfamethoxazole | *aadA2*, *aac(3)-IId*, *aph(3')-Ia*, *bla*_TEM-1_, *bla*_CTX-M-15_, *bla*_OXA-1_, *parC_S80I*, *aac(6')-Ib-cr5*, *gyrA_D87A*, *gyrA_S83F*, *tet*(A), *ramR_A19V*, *dfrA12* | NA |
| C1-173 | Cefepime, Cefotaxime, Ceftriaxone, Aztreonam, Ampicillin/Sulbactam, Amoxicillin/Clavulanic acid, Ciprofloxacin, Levofloxacin, Gentamicin, Trimethoprim/Sulfamethoxazole | *aadA2*, *aac(3)-IId*, *aph(3')-Ia*, *bla*_TEM-1_, *bla*_CTX-M-15_, *bla*_OXA-1_, *parC_S80I*, *aac(6')-Ib-cr5*, *gyrA_D87A*, *gyrA_S83F*, *ramR_A19V*, *dfrA12* | NA |
| C1-57 | Cefepime, Cefotaxime, Ceftriaxone, Aztreonam, Ampicillin/Sulbactam, Amoxicillin/Clavulanic acid, Ciprofloxacin, Levofloxacin, Gentamicin, Amikacin, Tigecycline, Trimethoprim/Sulfamethoxazole | *aadA2*, *aac(3)-IId*, *aph(3')-Ia*, *aph(6)-Id*, *aph(3'')-Ib*, *aadA1*, *armA*, *aph(4)-Ia*, *aac(3)-IVa*, *bla*_DHA-1_, *bla*_TEM-1_, *blaCTX-M-55*, *parC_S80I*, *qnrB4*, *gyrA_S83I*, *tet*(A), *ramR_A19V*, *toprJ1*, *tmexC1*, *tmexD1*, *dfrA12* | NA |
| C1-8 | Cefotaxime, Ceftriaxone, Ampicillin/Sulbactam, Amoxicillin/Clavulanic acid, Ciprofloxacin, Levofloxacin, Trimethoprim/Sulfamethoxazole | *aadA16*, *aph(3')-Ia*, *bla*_CTX-M-27_, *parC_S80I*, *aac(6')-Ib-cr5*, *gyrA_D87A*, *gyrA_S83F*, *ramR_A19V*, *tet*(D), *dfrA27* | *ybt10* |
| C4-30 | Ampicillin/Sulbactam, Amoxicillin/Clavulanic acid, Ciprofloxacin, Levofloxacin, Gentamicin, Trimethoprim/Sulfamethoxazole | *aadA2*, *aac(3)-IId*, *aph(3')-Ia*, *aph(4)-Ia*, *aac(3)-IVa*, *bla*_OXA-1_, *parC_S80I*, *aac(6')-Ib-cr5*, *gyrA_D87A*, *gyrA_S83F*, *tet*(A), *ramR_A19V*, *dfrA12* | *ybt10* |
| C4-33 | Cefepime, Cefotaxime, Ceftriaxone, Aztreonam, Ampicillin/Sulbactam, Amoxicillin/Clavulanic acid, Piperacillin/Tazobactam, Ciprofloxacin, Levofloxacin, Gentamicin, Trimethoprim/Sulfamethoxazole | *aadA2*, *aac(3)-IId*, *aph(3')-Ia*, *bla*_TEM-1_, *bla*_CTX-M-15_, *bla*_OXA-1_, *parC_S80I*, *aac(6')-Ib-cr5*, *gyrA_D87A*, *gyrA_S83F*, *tet*(A), *ramR_A19V*, *dfrA12* | NA |
| C5-20 | Cefepime, Cefotaxime, Ceftriaxone, Aztreonam, Ampicillin/Sulbactam, Amoxicillin/Clavulanic acid, Ciprofloxacin, Levofloxacin, Trimethoprim/Sulfamethoxazole | *aadA2*, *bla*_CTX-M-15_, *parC_S80I*, *gyrA_D87A*, *gyrA_S83F*, *ramR_A19V*, *dfrA12* | NA |
| C5-22 | Cefepime, Cefotaxime, Ceftriaxone, Aztreonam, Ampicillin/Sulbactam, Amoxicillin/Clavulanic acid, Piperacillin/Tazobactam, Ciprofloxacin, Levofloxacin, Gentamicin, Trimethoprim/Sulfamethoxazole | *aadA2*, *aph(3')-Ia*, *aph(4)-Ia*, *aac(3)-IVa*, *bla*_TEM-1_, *bla*_CTX-M-15_, *bla*_OXA-1_, *parC_S80I*, *aac(6')-Ib-cr5*, *gyrA_D87A*, *gyrA_S83F*, *tet*(A), *ramR_A19V*, *dfrA12* | NA |
| C6-104 | Cefotaxime, Ceftriaxone, Aztreonam, Ampicillin/Sulbactam, Amoxicillin/Clavulanic acid, Ciprofloxacin, Levofloxacin, Trimethoprim/Sulfamethoxazole | *aadA2*, *aph(3')-Ia*, *aph(6)-Id*, *aph(3'')-Ib*, *bla*_TEM-1_, *bla*_CTX-M-15_, *bla*_OXA-1_, *parC_S80I*, *aac(6')-Ib-cr5*, *gyrA_D87A*, *gyrA_S83F*, *tet*(A), *ramR_A19V*, *dfrA12* | NA |
| C6-114 | Cefepime, Cefotaxime, Ceftriaxone, Aztreonam, Ampicillin/Sulbactam, Amoxicillin/Clavulanic acid, Piperacillin/Tazobactam, Ciprofloxacin, Levofloxacin, Trimethoprim/Sulfamethoxazole | *aadA2*, *aph(3')-Ia*, *bla*_TEM-1_, *bla*_CTX-M-15_, *bla*_OXA-1_, *parC_S80I*, *aac(6')-Ib-cr5*, *gyrA_D87A*, *gyrA_S83F*, *tet*(A), *ramR_A19V*, *dfrA12* | *ybt15* |
| C6-128 | Cefepime, Cefotaxime, Ceftriaxone, Aztreonam, Ampicillin/Sulbactam, Amoxicillin/Clavulanic acid, Piperacillin/Tazobactam, Ciprofloxacin, Levofloxacin, Trimethoprim/Sulfamethoxazole | *aadA2*, *aph(3')-Ia*, *bla*_TEM-1_, *bla*_CTX-M-15_, *bla*_OXA-1_, *parC_S80I*, *aac(6')-Ib-cr5*, *gyrA_D87A*, *gyrA_S83F*, *tet*(A), *ramR_A19V*, *dfrA12* | *ybt15* |
| C6-131 | Cefepime, Cefotaxime, Ceftriaxone, Aztreonam, Ampicillin/Sulbactam, Amoxicillin/Clavulanic acid, Piperacillin/Tazobactam, Ciprofloxacin, Levofloxacin, Trimethoprim/Sulfamethoxazole | *aadA2*, *aph(3')-Ia*, *bla*_TEM-1_, *bla*_CTX-M-15_, *bla*_OXA-1_, *parC_S80I*, *aac(6')-Ib-cr5*, *gyrA_D87A*, *gyrA_S83F*, *tet*(A), *ramR_A19V*, *dfrA12* | *ybt15* |
| C6-135 | Cefepime, Cefotaxime, Ceftriaxone, Aztreonam, Ampicillin/Sulbactam, Amoxicillin/Clavulanic acid, Ciprofloxacin, Levofloxacin, Gentamicin | *aac(3)-IId*, *aph(3')-Ia*, *bla*_TEM-1_, *bla*_CTX-M-15_, *bla*_OXA-1_, *parC_S80I*, *aac(6')-Ib-cr5*, *qnrS1*, *gyrA_D87A*, *gyrA_S83F*, *tet*(A), *ramR_A19V* | NA |
| C6-35 | Cefepime, Cefotaxime, Ceftriaxone, Aztreonam, Ampicillin/Sulbactam, Amoxicillin/Clavulanic acid, Ciprofloxacin, Levofloxacin, Trimethoprim/Sulfamethoxazole | *aadA16*, *bla*_CTX-M-27_, *parC_S80I*, *aac(6')-Ib-cr5*, *gyrA_D87A*, *qnrB52*, *gyrA_S83F*, *ramR_A19V*, *dfrA27* | NA |
| C6-69 | Cefepime, Cefotaxime, Ceftriaxone, Aztreonam, Ampicillin/Sulbactam, Amoxicillin/Clavulanic acid, Piperacillin/Tazobactam, Ciprofloxacin, Levofloxacin, Trimethoprim/Sulfamethoxazole | *aadA2*, *aph(3')-Ia*, *bla*_TEM-1_, *bla*_CTX-M-15_, *bla*_OXA-1_, *parC_S80I*, *aac(6')-Ib-cr5*, *gyrA_D87A*, *gyrA_S83F*, *tet*(A), *ramR_A19V*, *dfrA12* | *ybt15* |
| C6-73 | Cefepime, Cefotaxime, Ceftriaxone, Aztreonam, Ampicillin/Sulbactam, Amoxicillin/Clavulanic acid, Ciprofloxacin, Levofloxacin, Gentamicin, Trimethoprim/Sulfamethoxazole | *aadA16*, *aac(3)-IId*, *aph(3')-Ia*, *aph(6)-Id*, *aph(3'')-Ib*, *bla*_TEM-1_, *bla*_CTX-M-3_, *parC_S80I*, *aac(6')-Ib-cr5*, *qnrS1*, *gyrA_S83I*, *tet*(A), *ramR_A19V*, *dfrA27* | *ybt10* |
| C6-76 | Cefepime, Cefotaxime, Ceftriaxone, Aztreonam, Ampicillin/Sulbactam, Amoxicillin/Clavulanic acid, Piperacillin/Tazobactam, Ciprofloxacin, Levofloxacin, Trimethoprim/Sulfamethoxazole | *aadA2*, *aph(3')-Ia*, *bla*_TEM-1_, *bla*_CTX-M-15_, *bla*_OXA-1_, *parC_S80I*, *aac(6')-Ib-cr5*, *gyrA_D87A*, *gyrA_S83F*, *tet*(A), *ramR_A19V*, *dfrA12* | *ybt15* |
| C6-89 | Cefepime, Cefotaxime, Ceftriaxone, Aztreonam, Ampicillin/Sulbactam, Amoxicillin/Clavulanic acid, Piperacillin/Tazobactam, Ciprofloxacin, Levofloxacin, Gentamicin, Tigecycline, Trimethoprim/Sulfamethoxazole | *aph(6)-Id*, *aph(3'')-Ib*, *aac(3)-IIe*, *bla*_CTX-M-27_, *bla*_TEM-1_, *bla*_CTX-M-15_, *bla*_OXA-1_, *parC_S80I*, *aac(6')-Ib-cr5*, *gyrA_D87A*, *qnrB1VgyrA_S83F*, *tet(A)*, *ramR_A19V*, *dfrA14* | *ybt10* |
| E1-165 | Imipenem, Meropenem, Cefepime, Cefotaxime, Ceftriaxone, Aztreonam, Ampicillin/Sulbactam, Amoxicillin/Clavulanic acid, Piperacillin/Tazobactam, Ciprofloxacin, Levofloxacin, Trimethoprim/Sulfamethoxazole | *aadA2*, *aac(3)-IId*, *aph(3')-Ia*, *aph(6)-Id*, *aac(6')-Ib3*, *aph(3'')-Ib*, *bla*_KPC-2_, *bla*_CTX-M-14_, *bla*_TEM-1_, *parC_S80I*, *gyrA_D87A*, *qnrB52*, *gyrA_S83F*, *tet*(A), *ramR_A19V*, *dfrA12* | *iuc5* |
| E1-38 | Cefepime, Cefotaxime, Ceftriaxone, Aztreonam, Ampicillin/Sulbactam, Amoxicillin/Clavulanic acid, Piperacillin/Tazobactam, Ciprofloxacin, Levofloxacin, Trimethoprim/Sulfamethoxazole | *aadA2*, *aph(3')-Ia*, *aadA1*, *bla*_TEM-1_, *bla*_CTX-M-15_, *bla*_OXA-1_, *parC_S80I*, *aac(6')-Ib-cr5*, *gyrA_D87A*, *gyrA_S83F*, *tet(A)*, *ramR_A19V*, *dfrA12* | NA |
